# The Autism Diagnostic Observation Schedule, Module 4: Application of the Revised Algorithms in an Independent, Well-Defined, Dutch Sample (n = 93)

**DOI:** 10.1007/s10803-015-2532-4

**Published:** 2015-08-30

**Authors:** Annelies de Bildt, Sjoerd Sytema, Harma Meffert, Jojanneke A. C. J. Bastiaansen

**Affiliations:** Department of Psychiatry, University Medical Center Groningen, University of Groningen, Groningen, The Netherlands; Accare University Center for Child and Adolescent Psychiatry, PO Box 660, 9700 AR Groningen, The Netherlands; Section on Affective and Cognitive Neuroscience, National Institutes of Health, Bethesda, MD USA; Interdisciplinary Center for Psychopathology and Emotion Regulation (ICPE), Department of Psychiatry, University Medical Center Groningen, University of Groningen, Groningen, The Netherlands; Social Brain Lab, Department of Neuroscience, University Medical Center Groningen, Groningen, The Netherlands; Forensic Psychiatric Clinic Dr. S. van Mesdag, Groningen, The Netherlands; Autism Team North Netherlands, Lentis, Groningen, The Netherlands

**Keywords:** Autism Spectrum Disorder, Schizophrenia, Psychopathy, Assessment, Classification, Adults

## Abstract

This study examined the discriminative ability of the revised Autism Diagnostic Observation Schedule module 4 algorithm (Hus and Lord in J Autism Dev Disord 44(8):1996–2012, 2014) in 93 Dutch males with Autism Spectrum Disorder (ASD), schizophrenia, psychopathy or controls. Discriminative ability of the revised algorithm ASD cut-off resembled the original algorithm ASD cut-off: highly specific for psychopathy and controls, lower sensitivity than Hus and Lord (2014; i.e. ASD .61, AD .53). The revised algorithm AD cut-off improved sensitivity over the original algorithm. Discriminating ASD from schizophrenia was still challenging, but the better-balanced sensitivity (.53) and specificity (.78) of the revised algorithm AD cut-off may aide clinicians’ differential diagnosis. Findings support using the revised algorithm, being conceptually conform the other modules, thus improving comparability across the lifespan.

## Introduction

The Autism Diagnostic Observation Schedule (ADOS, Lord et al. 1999) and more recently the ADOS-Second Edition (ADOS-2, Lord et al. 2012a, b) are widely used instruments in the diagnosis of Autism Spectrum Disorder (ASD). The ADOS(-2) aims to establish the presence of ASD across the whole life cycle (Lord et al. 2012a, b). The ADOS-2 has been developed from revisions of the ADOS, leading to revised algorithms and standardized severity scores for young age groups, ranging from toddlers to young adolescents [Toddler module (T) and modules 1–3]. These revised algorithms increase the comparability between modules, thereby improving the longitudinal comparison of ASD symptoms and severity. Initially, no revisions for adults and older adolescents were developed, which means that the comparability between the modules did not extend to module 4.

Additionally, until recently, research on the validity, reliability and value of the instrument had focused on younger age groups and was scarce in older adolescents and adults with fluent speech (module 4). In the manuals (Lord et al. 1999, 2012b), the reliability and validity of the original algorithm for module 4 was established based on 45 adolescents and adults [16 Autism (AD); 14 non-autism ASD, 15 non-spectrum], aged 10-40 years. The comparison group, with non-spectrum diagnoses, was a heterogeneous group. In 2011a, Bastiaansen and colleagues extended the research on module 4 with a study including 93 male adults (38 with ASD, 16 with psychopathy, 18 with schizophrenia and 21 controls, i.e. individuals without a clinical classification). The main findings indicated that the ADOS module 4 was a valid instrument. It was able to correctly classify the majority of participants (74.2 %), and higher scores on the ADOS predicted clinical ASD classifications. Based on group comparisons, the instrument discriminated ASD from psychopathy and controls. Distinguishing ASD from schizophrenia proved more difficult. The authors speculated that this was due to the behavioral overlap between the disorders (Frith and Happé 2005; Goldstein et al. 2002; Volkmar and Cohen 1991).

Recently, Hus and Lord (2014) developed a revised algorithm for module 4 in a large sample of adolescents and adults (393 participants with 437 administrations; mean age 21.56 years, SD 8.62, range 9.92–62.25). Consistent with the previous revisions of modules 1-3 and the development of module T (Lord et al. 2012a), the two-domain structure of the DSM-5 was found to be applicable in this sample. Accordingly, the revised module 4 algorithm consists of a Social Affect domain (SA) and a Repetitive Restricted Behaviors domain (RRB). The similarity of this structure to modules T and 1-3 corroborates the developmental continuity that the ADOS aims for. This continuity enables researchers as well as clinicians to examine developmental trajectories of ASD symptoms from toddlerhood into adolescence and adulthood.

Hus and Lord (2014) found good criterion-related validity in their sample, which included AD, ASD, and non-spectrum clinical referrals and clinical controls. Increasing scores on each domain, particularly RRB, predicted an increased probability of a clinical ASD classification. Based on the overall total score on the combined domains (SARRB), the classification of ASD (including AD and non-autism ASD) versus non-spectrum showed a sensitivity and specificity of above .80 in the total group.

Hus and Lord (2014) recommended further studies with the revised module 4 algorithm. Replication of the validity in independent samples is necessary before the field can begin to adopt the proposed revised algorithm in clinical practice and research. In the current study, we aimed to examine the diagnostic validity of the module 4 revised algorithm in the sample of Bastiaansen et al. (2011a), a diagnostically challenging sample including ASD, typical development, schizophrenia and psychopathy, i.e. other neurodevelopmental disorders that have behavioral overlap with ASD. The clinical presentation of schizophrenia overlaps with the clinical presentation of ASD, even though developmental trajectories may differ (Frith and Happé 2005; Goldstein et al. 2002; Volkmar and Cohen 1991). Specifically, negative symptoms in schizophrenia resemble the social symptoms seen in ASD (Frith and Happé 2005). Behavioral overlap in social communication also exists between psychopathy and ASD. Insensitivity or lack of empathy are characteristics that seem applicable to both diagnostic groups, although, again, they may originate from different sources (Bartels and Bruinsma 2008; Howlin 2000; Kohn et al. 1998). Thus, distinguishing these groups from ASD is challenging for clinicians. It is therefore important to determine whether information obtained from an observational assessment designed to assess ASD aides in this differential diagnosis. Thus, our objective was to examine the discriminative ability of the ADOS module 4 revised algorithm in our sample. Based on the results from Hus and Lord (2014), we hypothesized that the revised algorithm would better differentiate between the ASD and the non-ASD groups in this sample than the original algorithm.

## Methods

### Participants

The study sample was subject of an earlier study into the original module 4 algorithm by Bastiaansen et al. (2011a). The sample consisted of participants who applied for participation in two large neuroimaging studies into the neural basis of empathy (Bastiaansen et al. 2011b; Meffert et al. 2013). Both studies were approved by the Institutional Review Board of the University Medical Center Groningen, and all participants gave written informed consent.

The sample included 38 high-functioning, adult males with ASD (n = 8 AD, n = 17 Asperger Syndrome, n = 13 Pervasive Developmental Disorder-Not Otherwise Specified); 18, mainly outpatient, adult males with schizophrenia and predominantly negative symptomatology; 16 males with psychopathy from two forensic psychiatric clinics; and 21 males without any clinical classification, first-degree relatives with ASD, or a history of psychosis. In Table 1, age and IQ are presented for each diagnostic group. The groups did not differ in terms of age and IQ.

**Table 1:** Participant characteristics

	*n*	Age			IQ		
		Mean	SD	Range	Mean	SD	Range
ASD	38	31.82	11.24	18–66	101.14	14.67	73–133
Schizophrenia	18	37.00	10.73	19–61	89.17	13.89	68–112
Psychopathy	16	39.00	10.67	23–60	92.73	16.10	63–117
Controls	21	34.24	9.14	21–53	97.19	16.37	73–128

### Measures

#### Autism Diagnostic Observation Schedule (ADOS)

Psychologists who had obtained research reliability in administration and scoring administered the ADOS module 4 to all participants. This included all standard activities and the optional daily living items in order to obtain relevant background information. Due to the fact that the examiners recruited the participants themselves, they were not blind to previous clinical diagnoses at the time of assessment. The examiners scored the ADOS immediately after administration. These ‘live’ codes were used for enrollment of individuals with clinical ASD in the neuroimaging studies mentioned above. Additionally, all administrations were videotaped.

The current study was preceded by a study examining the inter-rater agreement on items, domains and classification of the ADOS module 4, as well as the validity (Bastiaansen et al. 2011a). For that study, we used consensus scores based on the videotapes. In order to increase comparability between the current and the former study we used these video based consensus scores in the current study as well. For these consensus scores, changing pairs of five trained and certified psychologists, who had reached research reliability, independently scored the interviews from the videotapes. The pairs included the examiner in the majority of cases. The examiner scored the interview from videotape again for the study, in order to create similar circumstances for both raters. The second rater was always blind to clinical diagnosis. Consensus scores were established based on the videotapes through a discussion in which the judgment of each rater was weighted equally, except for the items B1 ‘eye contact’ and B2 ‘facial expressions’. For these items the examiner’s opinion (based on live scores) was decisive when major disagreement occurred (i.e. 0 versus 2; this occurred in only two out of 93 administrations for B1, and never for B2). ADOS item consensus scores of 3 were recoded into 2 for the analyses.

As described, the revised algorithm consists of two domains, i.e. the Social Affect domain (SA) and the Restricted and Repetitive Behaviors (RRB) domain. The classification of the ADOS module 4 revised algorithm is based on the overall total score on the combination of the two domains (SARRB). In the current study, we refer to the domains as SA and RRB, and to the combination of both as the overall total score.

### Design and Analysis

In order to examine the discriminative ability of the module 4 revised algorithm in the current sample, the study focused on distinguishing ASD from schizophrenia, psychopathy and controls. Since the discriminative ability of an instrument depends on the nature of the control group, we have applied the analyses, where applicable, to the group with ASD, separately combined with each of the three other diagnostic groups.

First, we investigated the distribution of module 4 revised algorithm *item* scores in each diagnostic group.

Second, we applied a MANOVA analysis (using the GLM model in SPSS, version 22, with Bonferroni post hoc correction for multiple comparisons) to compare the mean module 4 revised algorithm *domain* scores (SA, RRB, overall total) of the diagnostic groups.

Third, the sensitivity of the algorithm was calculated for the ASD cut-off and the stricter AD cut-off, as well as the specificity of both cut-offs compared to schizophrenia, psychopathy and controls. The sensitivity indicates the proportion of participants *with* a clinical ASD classification that is correctly classified as ASD or AD by the ADOS module 4 revised algorithm, based on the overall total score. The specificity indicates the proportion of participants *without* a clinical ASD or AD classification that is classified as non-ASD by the ADOS module 4 revised algorithm.

Fourth, since the original and revised algorithms were applied to the same sample, we entered the ASD vs non-ASD outcomes and the AD vs non-AD outcomes of both algorithms into 2 × 2 crosstables. Based on these tables, we tested the outcome agreement for both the ASD and AD cut-offs with the McNemar test (McNemar 1947). We calculated McNemar’s statistic for the ASD sample, for which it represents a measure of the change in sensitivity between the algorithms. We also calculated McNemar’s statistic in the three other diagnostic groups, which indicates a change in specificity in these groups.

Although clinical practice needs single fixed cut-off points to classify an individual as ASD or AD, we also wished to investigate criterion-related validity by examining how the range of overall total scores was related to the clinical classification. Thus, fifth, a Receiver Operating Characteristic (ROC) analysis was performed in the patient sample only (to prevent inflation of results due to the inclusion of normal controls). Additionally, ROC analyses were performed separately for the ASD group in combination with each of the other three diagnostic groups (i.e. ASD and schizophrenia, ASD and psychopathy, and ASD and controls). A larger Area under the Curve (AuC) indicates a better overall level of agreement between the criterion (i.e. clinical ASD classification) and the instrument (i.e. ADOS module 4 overall total score), with a maximum value of 1.

Sixth, with logistic regressions we determined the predictive value of each of the domains for a clinical ASD classification, in order to investigate whether a specific domain would be predictive of ASD in comparison with the specific diagnostic groups. The Odds Ratio (OR) expresses the increase or decrease in odds of agreement between the domain scores and the clinical classification.

## Results

### Range of Scores on the ADOS Module 4 Revised Algorithm Items

Table 2 presents the frequencies of each score on each item of the revised algorithm for the four groups.

**Table 2:** Distribution of scores on Revised ADOS Module 4 algorithm items in four diagnostic groups

	ASD			Schizophrenia			Psychopathy			Controls		
Code	0	1	2	0	1	2	0	1	2	0	1	2
Algorithm items	*N* (%)	*N* (%)	*N* (%)	*N* (%)	*N* (%)	*N* (%)	*N* (%)	*N* (%)	*N* (%)	*N* (%)	*N* (%)	*N* (%)
*Social Affect*												
Unusual eye contact	26 (68.4)	–	12 (31.6)	14 (77.8)	–	4 (22.2)	16 (100)	–	0	21 (100)	–	0
Amount of social communication	13 (34.2)	8 (21.1)	17 (44.7)	9 (50.0)	7 (38.9)	2 (11.1)	15 (93.8)	1 (6.3)	0	15 (71.4)	6 (28.6)	0
Facial expressions	10 (26.3)	26 (68.4)	2 (5.3)	4 (22.2)	11 (61.1)	3 (16.7)	11 (68.8)	5 (31.3)	0	16 (76.2)	5 (23.8)	0
Quality of rapport	6 (15.8)	23 (60.5)	9 (23.7)	9 (50.0)	9 (50.0)	0	5 (31.3)	11 (68.8)	0	12 (57.1)	9 (42.9)	0
Communication own affect	9 (23.7)	14 (36.8)	15 (39.5)	6 (33.3)	8 (44.4)	4 (22.2)	9 (56.3)	5 (31.3)	2 (12.5)	11 (52.4)	7 (33.3)	3 (14.3)
Quality of social overtures	18 (47.4)	11 (28.9)	9 (23.7)	12 (66.7)	2 (11.1)	4 (22.2)	15 (93.8)	1 (6.3)	0	20 (95.2)	1 (4.8)	0
Conversation	25 (65.8)	7 (18.4)	6 (15.8)	15 (83.3)	3 (16.7)	0	16 (100)	0	0	21 (100)	0	0
Emphatic gestures	14 (36.8)	13 (34.2)	11 (28.9)	6 (33.3)	7 (38.9)	5 (27.8)	10 (62.5)	4 (25.0)	2 (12.5)	15 (71.4)	3 (14.3)	3 (14.3)
Quality of response	13 (34.2)	22 (57.9)	3 (7.9)	14 (77.8)	4 (22.2)	0	11 (68.8)	5 (31.3)	0	19 (90.5)	2 (9.5)	0
Insight	12 (31.6)	8 (21.1)	18 (47.4)	6 (33.3)	6 (33.3)	6 (33.3)	10 (62.5)	4 (25.0)	2 (12.5)	17 (81.0)	3 (14.3)	1 (4.8)
*Restricted Repetitive Behaviors*												
Speech abnormalities	21 (55.3)	13 (34.2)	4 (10.5)	11 (61.1)	6 (33.3)	1 (5.6)	15 (93.8)	1 (6.3)	0	18 (85.7)	3 (14.3)	0
Stereotyped language	17 (44.7)	19 (50.0)	2 (5.3)	15 (83.3)	2 (11.1)	1 (5.6)	12 (75.0)	4 (25.0)	0	19 (90.5)	2 (9.5)	0
Unusual sensory interest	37 (97.4)	1 (2.6)	0	17 (94.4)	1 (5.6)	0	16 (100)	0	0	21 (100)	0	0
Highly specific topics	30 (78.9)	5 (13.2)	3 (7.9)	14 (77.8)	4 (22.2)	0	16 (100)	0	0	21 (100)	0	0
Hand mannerisms	36 (94.7)	2 (5.3)	0	17 (94.4)	1 (5.6)	0	16 (100)	0	0	21 (100)	0	0

Three SA items received all three possible scores (0, 1 or 2) in all four groups, namely B5 ‘communication of own affect’, A10 ‘emphatic gestures’ and B7 ‘insight’. In the ASD group, all other SA items also received a score of 0, 1 or 2. While B2 ‘facial expressions’ and B10 ‘quality of social response’ received a score of 2 in less than 8 % of the ASD participants, the other SA items received a score of 2 in more than 15 % of the ASD participants. In the schizophrenia group, all SA items also received all possible scores except for the SA items B13 ‘quality of rapport’, A8 ‘conversation’ and B10 ‘quality of social response’, which never received a score of 2. In the psychopathy and control groups, scores of 0 and 1 were most prevalent.

With respect to the RRB items, the items D1 ‘unusual sensory interest’ and D2 ‘hand mannerisms’ did not receive a score of 2 in any of the four groups. In the ASD group, scores on the RRB items were predominantly 0 (44.7–97.4 %), but scores of 1 were relatively frequent for A2 ‘speech abnormalities’ (34.2 %), A4 ‘stereotyped language’ (50.0 %) and D4 ‘highly specific topics’ (13.2 %). These items also received occasional scores of 2. In the schizophrenia group, scores were also predominantly 0 with scores of 1 across all RRB items and occasional scores of 2 for A2 ‘speech abnormalities’ and A4 ‘stereotyped language’. In the psychopathy and control groups, three RRB items received scores of 0 only. Occasional 1’s were scored for the items A2 ‘speech abnormalities’ (6.3; 14.3 %) and A4 ‘stereotyped language’ (9.5; 25.0 %).

### Comparison of Groups on Domain Scores

As reported in Table 3, the MANOVA post hoc test showed that the mean domain scores of the module 4 revised algorithm of participants with ASD did not differ significantly from those with schizophrenia. The mean scores of participants with ASD were significantly higher than the mean scores of participants with psychopathy and controls.

**Table 3:** Mean domain scores on Revised ADOS Module 4 algorithm in four diagnostic groups

Domains	ASD (*n* = 38)	Schizophrenia (*n* = 18)	Psychopathy (*n* = 16)	Controls (*n* = 21)	*F*(3, 89)	Post-hoc tests
*Social Affect*						
Mean	8.84	6.28	3.00	2.43	15.95*	ASD > P***/C***
SD	5.04	3.88	1.83	2.13		S > C*
Range	0–17	2–16	0–7	0–8		
*Restricted Repetitive Behaviors*						
Mean	1.53	1.00	.31	.24	11.50*	ASD > P***/C***
SD	1.27	.77	.48	.44		
Range	0–4	0–2	0–1	0–1		
*Overall total score (SARRB)*						
Mean	10.37	7.28	3.31	2.67	18.40*	ASD > P***/C***
SD	5.75	4.13	2.02	2.27		S > P*/C**
Range	0–20	3–17	0–7	0–9		

* *p* < .05; ** *p* < .01; *** *p* < .001

### Sensitivity and Specificity of ADOS Original and Revised Algorithm Classifications

Table 4 presents the sensitivity and specificity of the revised and original algorithm in the current sample.

**Table 4:** Revised ADOS Module 4 sensitivity and specificity in Dutch adult sample

	Current sample				Hus and Lord (2014)	
	Sensitivity		Specificity		Sensitivity	Specificity
	ASD (*N* = 38)	Schiz (*N* = 18)	Psych (*N* = 16)	Contr (*N* = 21)	ASD (*N* = 437)	NS (*N* = 90)
*Revised algorithm*						
Overall total ASD (cut-off 8)	.61	.50	1.00	.95	.91	.82
Overall total AD (cut-off 10)	.53	.78	1.00	1.00	.79	.91
*Original algorithm*						
Met 3 domains ASD^a^	.55	.67	.94	.95	.90	.72
Met 3 domains AD^a^	.37	.89	1.00	1.00		

Overall total Social Affect and Restricted Repetitive Behaviors

*ASD* Autism Spectrum Disorder*, AD* autism, *Schiz* schizophrenia, *Psych* psychopathy, *Contr* controls, *NS* nonspectrum

^a^Met or exceeded cut-offs for ASD or AD on social, communication and social + communication domains

Sensitivity of the ADOS revised algorithm classifications based on the ASD cut-off and on the AD cut-off were .61 and .53, respectively. This means that 61 % of the individuals with a clinical ASD diagnosis exceeded the ADOS cut-off for ASD and 53 % exceeded the ADOS cut-off for AD on the revised algorithm. The sensitivity of the revised algorithm resembled the original algorithm for the ASD cut-off (.55) and was higher for the original algorithm AD cut-off (.37).

Specificity varied considerably, depending on the included diagnostic comparison group. Specificity was relatively low when including the schizophrenia group; 50 % of the individuals with a clinical schizophrenia classification exceeded the cut-off for ASD on the revised ADOS algorithm (n = 9), compared to 33 % on the original algorithm (n = 6). With the stricter cut-off for AD, this decreased to 22 % (n = 4) on the revised and 11 % (n = 2) on the original algorithm. These findings indicate that the specificity when distinguishing between ASD and schizophrenia did not improve with the revised algorithm. For the psychopathy and the control groups, specificity approached 1 for both algorithms. This means that individuals with psychopathy and controls are (almost) never classified by the ADOS as an ASD case.

Additionally reported in Table 4 are the sensitivity and specificity as observed by Hus and Lord (2014). The sensitivity for ASD found in the current sample did not reach the levels reported by Hus and Lord (2014). The specificity with respect to psychopathy and controls resembled the specificity the sample of Hus and Lord (2014). For schizophrenia, the specificity was lower than the one reported by Hus and Lord (2014).

Table 5 illustrates the cases on which the original algorithm and the revised algorithm disagreed on ASD or AD classification.

**Table 5:** ADOS Module 4 agreement on ASD classification, between original and revised algorithm (n = 93)

	Original algorithm			Original algorithm	
Revised algorithm	ASD	Non-ASD	Revised algorithm	AD	Non-AD
*Agreement in ASD sample (n* *=* *38)*					
ASD	19	4	AD	14	6
Non-ASD	2	13	Non-AD	0	18
*Agreement in non-ASD sample (n* *=* *55)*					
ASD	7^a^	3^b^	AD	2	2^b^
Non-ASD	1^c^	44	Non-AD	0	51

^a^Schizophrenia, *n* = 6; TD *n* = 1

^b^Schizophrenia

^c^Psychopathy

This table shows what non-ASD cases exceeded ASD or AD cut-off (sensitivity) and what ASD cases did not reach ASD or AD cut-off (specificity). The algorithms disagreed on ten cases based on the ASD cut-off. Three cases exceeded the cut-off for ASD on the original algorithm but not on the revised algorithm, two with clinical ASD, one with psychopathy. Conversely, seven cases exceeded the ASD cut-off on the revised algorithm but not on the original algorithm, four with clinical ASD and three with schizophrenia. Based on the stricter cut-off for AD, the algorithms disagreed on eight cases, all of which exceeded AD cut-off on the revised but not on the original algorithm. Six of these eight had a clinical ASD classification, the two others were from the schizophrenia group. This illustrates the increase in sensitivity of the AD cut-off of the revised algorithm compared to the original algorithm, but also shows that the sensitivity and specificity of the ASD cut-off remain fairly the same, even with other cases exceeding the ASD cut-off.

The McNemar change statistic did not show a significant difference between the classification outcomes of the ASD cut-offs of both algorithms in the ASD sample (Χ^2^ = .167, *p* = .683). However, the classification outcomes based on the AD cut-off of both algorithms did differ significantly between the algorithms in the ASD sample (Χ^2^ = 4.167, *p* = .041). The revised algorithm classified more clinical ASD cases as AD than the original algorithm, and the McNemar statistic indicates an increase in sensitivity for the stricter AD cut-off of the revised algorithm compared to the stricter AD cut-off of the original algorithm.

In the non-ASD sample, the classification outcomes did not differ between the two algorithms in the schizophrenia group (ASD cut-off: Χ^2^ = .248, *p* = .248; AD cut-off: Χ^2^ = .500, *p* = .480), the psychopathy group (ASD cut-off: Χ^2^ = .000, *p* = 1.000) or the control group (ASD cut-off: Χ^2^ = .000, *p* = 1.000). This indicates that specificity for none of the groups changed with the revision of the algorithm. In the psychopathy group and the control group, the McNemar statistic could not be calculated for the AD cut-off. In both groups, only one cell of the crosstable was filled, since none of the participants exceeded AD cut-off on either of the algorithms.

### ROC Analyses

ROC analyses were applied for the overall total score, with a clinical ASD classification as the criterion. In the sample including ASD, schizophrenia and psychopathy, but excluding controls, the AuC was .75. This indicates adequate criterion-related validity when the full range of scores is taken into account. The AuC was .66 in the sample of ASD and schizophrenia, .86 in the sample of ASD and psychopathy, and .88 in the sample of ASD and controls.

### Logistic Regressions

With logistic regression in the total sample, the predictive value of each domain for a clinical ASD classification was investigated. For SA, the OR was 1.22 (95 % CI 1.07–1.39; *p* = .004), for RRB the OR was 1.97 (95 % CI 1.05–3.70; *p* = .034). This means that each additional point on the SA domain increased the odds of a clinical ASD classification with a factor 1.22 and each additional point on RRB with a factor 1.97.

None of the domains had predictive value for discriminating between ASD and schizophrenia. That is, in the group including only ASD and schizophrenia in the analysis, the OR’s were slightly lower and not significant (SA OR 1.10, 95 % CI .975–1.26, *p* = .207; RRB OR 1.34, 95 % CI .709–2.53, *p* = .369). For discriminating between ASD and psychopathy, the SA domain (OR 1.33, 95 % CI 1.03–1.72, *p* = .030) but not the RRB domain (OR 3.34, 95 % CI .93–12.01, *p* = .065) had predictive value. For the discrimination between ASD and controls, both the SA and RRB domain had predictive value (SA OR 1.38, 95 % CI 1.07–1.78, *p* = .013; RRB OR 4.35, 95 % CI 1.14–16.54, *p* = .031). Thus, the analyses in the separate groups show that the predictive value is affected by the comparison group.

## Discussion

Recently, Hus and Lord (2014) developed a revised algorithm for the ADOS module 4 in a large sample of adolescents and adults. Replication of the discriminative validity in independent samples is important for application of the revised algorithm in clinical practice and research. The current study therefore aimed to confirm the discriminative ability of the ADOS module 4 revised algorithm (Hus and Lord; 2014) in an independent sample of 93 Dutch adult males with ASD, compared to a challenging non-ASD sample including individuals with schizophrenia, psychopathy and controls.

Based on the findings of Hus and Lord (2014), and the items included in the revised algorithm, we hypothesized that the revised module 4 algorithm would better differentiate between ASD and non-ASD in the current sample than the original algorithm. This hypothesis could partially be confirmed based on the current findings. Our main finding is that the original algorithm does not outperform the revised algorithm. On the contrary, the revised algorithm had a few advantages over the original algorithm. First we observed a small but significant gain in sensitivity for the revised algorithm AD cut-off, compared to the original algorithm AD cut-off. Second, there was a small improvement in discriminating schizophrenia from ASD, but only when the AD cut-off was applied. Third, when discriminating schizophrenia from ASD, the AD cut-off provides a better balance between sensitivity and specificity in the revised algorithm.

The first advantage was the small but significant gain in sensitivity based on the revised algorithm AD cut-off, compared to the original algorithm AD cut-off, even though it was still lower than reported by Hus and Lord (2014). The sensitivity based on the ASD cut-off was comparably low for the revised and the original algorithm, and was also lower than reported by Hus and Lord (2014). This indicates that the ASD cut-off on the ADOS module 4 revised algorithm tended to overlook part of the individuals with a clinical classification of ASD in the current sample.

Several characteristics of the current research sample may have contributed to a lower sensitivity compared to that found in the sample of Hus and Lord (2014). First of all, as Bastiaansen et al. (2011a) already pointed out, the high level of functioning of the ASD participants may have been of importance; all participants were able to partake in an extensive functional magnetic resonance research project. Therefore, the adults with ASD may have presented with less clear-cut or suppressed ASD symptoms during the ADOS interview. This explanation may seem somewhat at odds with the fact that the reported sensitivity for individuals with average to above average IQs reported by Hus and Lord (2014) was still considerably higher (.87) than that reported in the current study. However, it is important to keep in mind that high IQ does not necessarily equate high functioning. Additionally or alternatively, the differences in age range between the two samples may have played a role. In the sample of Hus and Lord (2014), only 9 participants were 40 years or older (i.e. 2.3 % of the 393 participants) whereas the current sample contained 32 participants aged 40 or older (i.e. 34.4 %). Additionally, 23 participants in the current sample were 30 through 39 years old (24.7 %). Perhaps our ‘older’ adults (i.e. over 30 or 40 years) showed different expressions of their ASD symptoms that differentiated them from younger adults and adolescents. Thirdly, rating the ADOS from videotapes compared to live administrations may have decreased the sensitivity, in that more subtle behaviors may have been less well observable from these recordings. However, this does not seem likely, as there was little disagreement on the items that are most difficult to score from screen, i.e. eye contact and facial expressions.

The second advantage was the improved discriminative ability of the revised algorithm AD cut-off over the original algorithm AD cut-off, when distinguishing ASD from schizophrenia specifically. Unfortunately, no clear gain was found when the ASD cut-off was used. This is likely due to the fact that the scores on the SA and RRB domains and the overall total score did not differ significantly between those two groups. Indeed, the AuC statistic demonstrated a low probability that a randomly chosen participant with ASD had a higher score on the instrument than a randomly chosen participant with schizophrenia. Logistic regression also indicated that neither the SA nor the RRB domain contributed to the clinical ASD classification in the combined ASD and schizophrenia group. Additionally, specificity of the ASD cut-off was rather low; the ASD cut-off on the revised algorithm identified 50 % of the individuals with clinical diagnosis of schizophrenia as ASD. However, applying the stricter AD cut-off showed a much higher specificity of .78 in the sample with schizophrenia compared to the ASD cut-off. This value is of acceptable level and approaches the specificity of module 3 (.84, Gotham et al. 2007). In the current schizophrenia sample, the number of inaccurately classified individuals decreased from nine (cut-off for ASD) to four when using the cut-off for AD. In other words, the stricter AD cut-off seems essential in order to reduce the number of false positives in the schizophrenia group. It is important to note that applying the stricter cut-off for AD will inevitably lead to a decrease in sensitivity. The higher specificity for the AD cut-off is in line with the fact that Hus and Lord (2014) added the AD cut-off for researchers in order to increase specificity (e.g. inclusion of definite cases). The single ASD cut-off was provided in order to be consistent with the diagnostic criteria of the DSM-5 in which AD is no longer differentiated from ASD (Hus and Lord 2014).

A third advantage of the revised over the original algorithm is the better balance between sensitivity and specificity when distinguishing ASD from schizophrenia, when the AD cut-off is applied (revised algorithm: sensitivity .53 and specificity .78; original algorithm: sensitivity .37 and specificity .89), even though the actual specificity of the AD cut-off in the sample with schizophrenia is lower than for the original algorithm. This is an important finding, since an instrument with high specificity but low sensitivity will miss clinically classified cases, whereas high sensitivity with low specificity indicates a tendency to be over inclusive. Moreover, despite the loss in specificity compared to the original algorithm, there is a significant improvement in AD classification outcome on the revised algorithm. Based on the actual behavioral overlap between ASD and schizophrenia, perfect sensitivity and specificity cannot be anticipated.

Bastiaansen et al. (2011a) already showed that only three module 4 items discriminated between ASD and schizophrenia: ‘stereotyped language’, ‘quality of social response’ and ‘quality of rapport’. Since these three items are already included in the module 4 revised algorithm, adding or omitting others would probably not be of any value for distinguishing ASD from schizophrenia in the current sample. The difficulties in discriminating ASD from schizophrenia likely reflect the actual behavioral overlap between ASD and schizophrenia, specifically when marked negative symptoms are present (Frith and Happé 2005; Sheitman et al. 2004). Bastiaansen et al. (2011a) reported that the degree of negative symptomatology correlated significantly with ADOS scores, in particular with items resembling negative symptoms in their sample. Due to this actual behavioral resemblance, the discriminative difficulty is probably not instrument specific, but inherent to these disorders.

The revised algorithm was equally well able to discriminate between ASD and psychopathy or controls as the original algorithm. Individuals with ASD had significantly higher scores on the ADOS revised algorithm than individuals with psychopathy or controls, on both domains and the overall total score. Additionally, specificity was high (.95–1). That is, individuals with psychopathy and controls (almost) never exceeded ADOS cut-off for ASD or AD. On top of that, the criterion-related validity was good as indicated by high AuC statistics. With respect to the domains, in the comparisons between i) ASD and psychopathy and ii) ASD and controls, the SA domain was important, with a significant increase in the odds of a clinical ASD classification for each additional point on the SA domain. In the control group, increasing scores on RRB also increased the odds of a clinical ASD classification. Unexpectedly, in the psychopathy group, the RRB domain did not reach statistical significance. We would have expected the RRB domain to increase the odds of a clinical ASD classification also in the comparison with psychopathy, since RRBs are not symptomatic of psychopathy. The comparable ORs in the psychopathy group and the controls, the item score distribution and the *p* value (approaching *p* < .05) suggest that insufficient statistical power may have led to the finding of a pattern that is not similar to that reported by Hus and Lord (2014). Overall, the current findings seem to confirm the value of both domains of the ADOS module 4 revised algorithm.

### Limitations

The current study included disorders chosen for their partial symptom overlap. This overlap maximally challenges clinicians in their diagnostic process, and we aimed to determine whether the ADOS module 4 revised algorithm aides in this differential diagnostic process. However, the relatively small sample size of each diagnostic category warrants careful interpretation of the findings on discriminative ability of the ADOS module 4 revised algorithm.

In addition, the administrators of the ADOS were not blind to the clinical diagnosis of the participants at the time of assessment. This may have influenced the administration and evaluation of behavior. Also, the fact that raters in the study scored the ADOS from video and used those video-based scores for consensus, is not in line with the standardization of the ADOS, since the interview should be scored immediately after and based on the live administration. In the current study, the consensus score from videotapes was chosen to increase comparability with the previous study in the same sample (Bastiaansen et al. 2011a). As explained in the methods section, the former study was based on consensus scores.

## Conclusion

The current study showed that the discriminative ability of the ASD cut-off on the ADOS revised algorithm resembled that of the ASD cut-off of the original algorithm: highly specific for psychopathy and controls, lower sensitivity compared to Hus and Lord (2014). The gain is found in the increased sensitivity for ASD based for the revised algorithm AD cut-off compared to the original algorithm. This is essential for a better discrimination between ASD and schizophrenia, which improved as well with the revised algorithm AD cut-off. Additionally, the better, acceptable balance between sensitivity and specificity of the AD cut-off on the revised algorithm may be valuable in the differential diagnostic process of clinicians trying to differentiate between ASD and schizophrenia.

The findings in this study corroborate the use of the ADOS module 4 revised algorithm, with the great advantage of improving the comparability with the previously revised algorithms. However, the findings also still corroborate earlier recommendations to combine the ADOS with other instruments and sources of information in a multi-modal assessment for diagnostic purposes (Bastiaansen et al. 2011a; Lord et al. 2012a, b).
